# Human-rabbit Hybrid Translation System to Explore the Function of Modified Ribosomes

**DOI:** 10.21769/BioProtoc.4714

**Published:** 2023-07-05

**Authors:** Eriko Matsuura-Suzuki, Hirotaka Toh, Shintaro Iwasaki

**Affiliations:** 1 RNA Systems Biochemistry Laboratory, RIKEN Cluster for Pioneering Research, Wako, Saitama 351-0198, Japan; 2 Department of Computational Biology and Medical Sciences, Graduate School of Frontier Sciences, The University of Tokyo, Kashiwa, Chiba 277-8561, Japan

**Keywords:** In vitro translation, Ribosome, Translation elongation, Post-translational modification, Rabbit reticulocyte lysate (RRL), Hybrid translation system, Dual luciferase assay, Modified ribosome

## Abstract

*In vitro translation systems* are a useful biochemical tool to research translational regulation. Although the preparation of translation-competent cell extracts from mammals has often been a challenge, the commercially available rabbit reticulocyte lysate (RRL) is an exception. However, its valid use, investigating the mechanism of translation machinery such as ribosomes in RRL, presents an analytic hurdle. To overcome this issue, the hybrid translation system, which is based on the supplementation of purified human ribosomes into ribosome-depleted RRL, has been developed. Here, we describe the step-by-step protocol of this system to study translation driven by ribosomes lacking post-translational modifications of the ribosomal protein. Moreover, we combined this approach with a previously developed reporter mRNA to assess the processivity of translation elongation. This protocol could be used to study the potency of heterologous ribosomes.

## Background

In addition to transcription, much of gene expression is regulated at the protein synthesis step. Translational regulation is central to diverse cellular and organismal processes, such as development, differentiation, memory, viral infection, tumorigenesis, stress response, and the cell cycle. A wide variety of tools have been developed to monitor the translational output in cells [reviewed in Iwasaki and Ingolia (2017)]. These include fluorescence-based techniques [e.g., fluorescent noncanonical amino acid tagging (Dieterich et al., 2010)], mass spectrometry–based approaches [e.g., pulsed stable isotope labeling by amino acids in cell culture (Schwanhäusser et al., 2009)], ribosome profiling (Ingolia et al., 2009), and single-molecule imaging by Sun tags and spaghetti monster tags (Morisaki et al., 2016; Wang et al., 2016; Wu et al., 2016; Yan et al., 2016). However, for the detailed exploration of molecular mechanisms, in vitro translation remains a powerful and valuable methodology.

Although purified factors could serve for the biochemical reconstitution of protein synthesis processes (Pestova et al., 1998; Shimizu et al., 2001; Pestova and Hellen, 2003; Alkalaeva et al., 2006; Pisarev et al., 2007; Machida et al., 2018; Yokoyama et al., 2019; Abe et al., 2020), the preparation of individual factors is not an easy task. Thus, in vitro translation with cell extracts has become an attractive and useful strategy (Gregorio et al., 2019). For mammals, lysate-based in vitro translation systems were developed with a variety of cell types, such as CHO (Brödel et al., 2014), HEK293 (Fritz et al., 2018), HeLa (Molla et al., 1991; Bergamini et al., 2000; Witherell, 2001; Thoma et al., 2004; Mikami et al., 2006; Rakotondrafara and Hentze, 2011), and Krebs-2 (Kerr et al., 1966; Mathews and Korner, 1970; Svitkin and Agol, 1978; Svitkin and Sonenberg, 2004 and 2007). Moreover, rabbit reticulocyte lysate (RRL) (Hunt and Jackson, 1974; Pelham and Jackson, 1976; Jackson and Hunt, 1983) has been widely used due to its commercial availability and high translational capacity. However, RRL struggles in depleting proteins by gene knockout or knockdown. Although immunodepletion by antibodies (Rakotondrafara and Hentze, 2011) has been used for this purpose, this technique depends on the efficacy, specificity, and availability of the antibodies for proteins of interest.

This barrier similarly applies to the investigation of ribosomes. Given the growing evidence of modifications of ribosomal RNA (Penzo et al., 2015; Erales et al., 2017; Taoka et al., 2018), ribosomal proteins (Simsek and Barna, 2017), and the heterologous composition of the constituents (Emmott et al., 2019), specialized ribosomes with diverse functions have been proposed (Genuth and Barna, 2018a and 2018b; Guo, 2018). Although ribosome profiling and proteome analysis in vivo provide global views of the function of specialized ribosomes (Ferretti and Karbstein, 2019), biochemical assay systems complement those approaches.

A technique termed the *hybrid translation system*, which is based on the exchange of ribosomes, is an alternative option in such studies (Panthu et al., 2015; Penzo et al., 2016; Erales et al., 2017; Trainor et al., 2021). Given that ultracentrifugation sediments the ribosome into the pellet, RRL can be prepared as ribosome-free but translation-competent only when the ribosomes are replenished. By supplementation with purified ribosomes of interest, for example from factor-mutated human cell lines, this system makes it possible to test the function of ribosomes in the context of active protein synthesis.

Moreover, the designed reporter enables researchers to investigate the effect of specific codons or RNA sequences along mRNA on translation elongation (Kisly et al., 2018 and 2021). The reporter consists of the fusion of *Renilla* and firefly luciferases (Rluc and Fluc) and measures the difference in the speeds of Rluc and Fluc synthesis to determine the ribosome elongation rate. Whereas downstream firefly luciferase detection requires complete reporter protein translation (as a whole process of initiation, elongation, and termination), upstream *Renilla* luciferase can be monitored even in the middle of translation elongation. Thus, the synthesis rate difference between the two luciferases provides a near proxy for elongation efficacy. The insertion of a sequence of interest between the luciferases allows a motif-specific effect on ribosome processivity.

Here, we describe the step-by-step method for the concomitant use of two experimental setups. This protocol includes four major steps: 1) the preparation of ribosome-depleted RRL; 2) the purification of ribosomes from HEK293 cells; 3) the preparation of reporter mRNAs; and 4) the translation reaction. The first two steps were based on an earlier study (Panthu et al., 2015). The design of the reporter mRNA followed the work reported by Tamm and colleagues (Kisly et al., 2021). The combination of the two approaches allowed us to study the effect of the histidine methylation of ribosomal protein uL3 (or RPL3) (Matsuura-Suzuki et al., 2022). Concretely, we delineated the results of ribosomes purified from naïve HEK293T cells and ribosomes purified from cells deficient for *METTL18*, the enzyme responsible for the methylation of uL3 on His245 (Małecki et al., 2021; Matsuura-Suzuki et al., 2022). In the reporter mRNA, we inserted Tyr repeats between the two luciferases to monitor the ribosome processivity on those codons. Similar applications of this methodology will expand our understanding of the regulatory mechanisms of decorated or specialized ribosomes along diverse codon/RNA element contexts.

## Materials and reagents

1. Pipette, 10 mL, graduated 1/10 mL, sterile, paper-plastic packaging, single packed (Greiner Bio-One, catalog number: 607180)
2. Pipette, 25 mL, graduated 2/10 mL, sterile, paper-plastic packaging, single packed (Greiner Bio-One, catalog number: 760180)
3. 10 μL, long, graduated, filter tip with system rack (pp), sterilized (WATSON, catalog number: 1252P-207CS)
4. 20 μL hyper filter tip with system rack (pc), sterilized (WATSON, catalog number: 125-20S)
5. 200 μL hyper filter tip with system rack (pc), sterilized (WATSON, catalog number: 125-200S)
6. 1,000 μL, long, graduated, filter tip with system rack (pc), sterilized (WATSON, catalog number: 124-1000S)
7. TipXL box (IKA Works, catalog number: 0020017832)
8. Tube, 15 mL, pp, 17/120 mm, natural, sterile, 20 pcs/bag (Greiner Bio-One, catalog number: 188271-013)
9. Labcon SuperClear 1.5 mL screw cap microcentrifuge tubes (Thermo Fisher Scientific, catalog number: 3611-870-000)
10. 11 mm diameter Delrin tube adapter (Beckman Coulter, catalog number: 393238)
11. DNA LoBind tubes, 1.5 mL (Eppendorf, catalog number: 0030108418)
12. Nunc EasYDishes, 100 mm (Thermo Fisher Scientific, catalog number: 150466)
13. 3.2 mL, open-top thick wall polycarbonate tube, 13 mm × 56 mm (Beckman Coulter, catalog number: 362305)
14. 0.2 mL 8-strip PCR tube and cap (NIPPON Genetics, catalog number: FG-028FC)
15. Screw cap tube, 5 mL (L × Ø): 57 mm × 15.3 mm, PP (SARSTEDT, catalog number: 60.558.001)
16. Corning 96-well white flat bottom polystyrene not treated microplate, 25 per bag, without lid, nonsterile (Corning, catalog number: 3912)
17. Rabbit reticulocyte lysate (RRL), nuclease-treated (Promega, catalog number: L4960, stored at -80°C)
18. DMEM, high glucose, GlutaMAX supplement (Thermo Fisher Scientific, catalog number: 10566016, stored at 4°C)
19. Fetal bovine serum (FBS) (MERCK, catalog number: F7524, stored at -20 °C)
20. HEK293T (RIKEN BRC, catalog number: RCB2202)
21. *METTL18* KO HEK293T (Matsuura-Suzuki et al., 2022)
22. D-PBS(-) without Ca and Mg, liquid (Nacalai Tesque, catalog number: 14249-24, stored at room temperature)
23. 1 M HEPES-KOH buffer solution (pH 7.5) (Nacalai Tesque, catalog number: 15639-84, stored at room temperature)
24. Potassium acetate (KOAc), nuclease and protease tested (Nacalai Tesque, catalog number: 28434-25, stored at room temperature)
25. Magnesium acetate tetrahydrate (MgOAc_2_·4H_2_O), nuclease and protease tested (Nacalai Tesque, catalog number: 20849-32, stored at room temperature)
26. Dithiothreitol (DTT), nuclease tested (Nacalai Tesque, catalog number: 14128-62, stored at 4 °C)
27. UltraPure DNase/RNase-free distilled water (Thermo Fisher Scientific, catalog number: 10977-015, stored at room temperature)
28. Sucrose, ultra pure (FUJIFILM, catalog number: 198-13525, stored at room temperature)
29. 5 M sodium chloride (NaCl) solution (Nacalai Tesque, catalog number: 06900-14, stored at room temperature)
30. KCl (2 M), RNase-free (Thermo Fisher Scientific, catalog number: AM9640G, stored at room temperature)
31. 1 M magnesium chloride (MgCl_2_) solution, sterile filtered (Nacalai Tesque, catalog number: 20942-34, stored at room temperature)
32. 2-Mercaptoethanol, nuclease tested (Nacalai Tesque, catalog number: 21438-82, stored at 4 °C)
33. Qubit RNA BR Assay kit (Thermo Fisher Scientific, catalog number: Q10210) (accompanied with Qubit RNA BR buffer, Qubit RNA BR reagent, and 0.5 mL PCR tubes)
34. 1 M Tris-HCl solution (pH 6.8) (BioVision, catalog number: 2106-100, stored at room temperature)
35. UltraPure SDS solution, 10% (Thermo Fisher Scientific, catalog number: 15553-035, stored at room temperature)
36. Glycerol (Nacalai Tesque, catalog number: 17018-25, stored at room temperature)
37. Bromophenol blue (Nacalai Tesque, catalog number: 05808-61, stored at room temperature)
38. BLUE Star prestained protein ladder (NIPPON Genetics, catalog number: MWP03-8, stored at -20 °C)
39. SuperSep Ace, 5%–20%, 17 well (FUJIFILM, catalog number: 194-15021, stored at 4 °C)
40. Tris(hydroxymethyl)aminomethane (Nacalai Tesque, catalog number: 35406-91, stored at room temperature)
41. Glycine (Nacalai Tesque, catalog number: 17109-35, stored at room temperature)
42. Sodium lauryl sulfate (Nacalai Tesque, catalog number: 31606-75, stored at room temperature)
43. GelCode blue stain (Thermo Fisher Scientific, catalog number: 24590, stored at room temperature)
44. Methanol (FUJIFILM, catalog number: 131-01826, stored at room temperature)
45. Acetic acid (Nacalai Tesque, catalog number: 00212-85, stored at room temperature)
46. psiCHECK2-Y0× (Matsuura-Suzuki et al., 2022)
47. psiCHECK2-Y39× (Matsuura-Suzuki et al., 2022)
48. Primer 1, 5′-TGACTAATACGACTCACTATAGG-3′ dissolved in TE (eurofins, stored at -20 °C) (Matsuura-Suzuki et al., 2022)
49. Primer 2, 5′-TGTATCTTATCATGTCTGCTCGAA-3′ dissolved in TE (eurofins, stored at -20 °C) (Matsuura-Suzuki et al., 2022)
50. TE buffer solution (pH 8.0), nuclease and protease tested (Nacalai Tesque, catalog number: 32739-31, stored at room temperature)
51. PrimeSTAR Max DNA polymerase (TaKaRa, catalog number: R045A, stored at -20 °C)
52. 0.5 M EDTA (pH 8.0) (NIPPON GENE, catalog number: 311-90075, stored at room temperature)
53. Agarose for ≥ 1 kbp fragment (Nacalai Tesque, catalog number: 01163-05, stored at room temperature)
54. 10× loading buffer (TaKaRa, catalog number: 9157, stored at room temperature)
55. 1 kb DNA Ladder (New England BioLabs, catalog number: N3232S, stored at -20 °C)
56. GreenView nucleic acid gel stain, 10,000× in water (RELYON, catalog number: N100, stored at 4 °C)
57. NucleoSpin Gel and PCR Clean-up (MACHEREY-NAGEL, catalog number: 740609.50, stored at room temperature)
58. T7-Scribe Standard RNA IVT kit (CELLSCRIPT, catalog number: C-AS3107, stored at -20 °C) (accompanied with 10× T7-Scribe transcription buffer, 100 mM ATP, 100 mM CTP, 100 mM UTP, 100 mM DTT, 40 U/μL ScriptGuard RNase inhibitor, T7-Scribe enzyme solution, RNase-free water, and DNase I)
59. Agencourt RNAClean XP (Beckman Coulter, catalog number: A63987, stored at 4 °C)
60. Ethanol (99.5) for molecular biology (FUJIFILM, catalog number: 054-07225, stored at room temperature)
61. ScriptCap m^7^G capping system (CELLSCRIPT, catalog number: C-SCCE0625, stored at -20 °C)
62. ScriptCap 2′-*O*-methyltransferase kit (CELLSCRIPT, catalog number: C-SCMT0625, stored at -20 °C)
63. A-Plus Poly(A) Polymerase Tailing kit (CELLSCRIPT, catalog number: C-PAP5104H, stored at -20 °C)
64. RNA 1000 kit (SHIMADZU, catalog number: 292-27913-91, stored at -20 °C and 4 °C), supplied with separation buffer and marker solution
65. SYBR Green II RNA gel stain, 10,000× concentrate in DMSO (Thermo Fisher Scientific, catalog number: S7564, stored at -80 °C)
66. RNA 6000 ladder (Thermo Fisher Scientific, catalog number: AM7152, stored at -80 °C)
67. Formamide (Nacalai Tesque, catalog number: 16229-95, stored at room temperature)
68. Amino acid mixtures (Promega, catalog number: L4461, stored at -80 °C)
69. Recombinant RNase inhibitor (TaKaRa, catalog number: 2313A, stored at -20 °C)
70. Dual-Luciferase Reporter Assay System (Promega, catalog number: E1910, stored at -20 °C), supplied with passive lysis buffer, 5× (Promega, catalog number: E1941, stored at -20 °C)
71. Liquid nitrogen
72. DMEM supplemented with FBS (500 mL) (see Recipes)
73. 1 M KOAc (5 mL) (see Recipes)
74. 1 M MgOAc_2_ (1 mL) (see Recipes)
75. 1 M DTT (5 mL) (see Recipes)
76. Buffer R (10 mL) (see Recipes)
77. Sucrose cushion solution (10 mL for eight samples) (see Recipes)
78. Buffer R2 (5 mL) (see Recipes)
79. 2× Laemmli sample buffer (see Recipes)
80. 10× SDS-PAGE running buffer (see Recipes)
81. 1× SDS-PAGE running buffer (see Recipes)
82. Gel fixation buffer (see Recipes)
83. 50× TAE (1 L) (see Recipes)
84. 1% agarose gel (100 mL) (see Recipes)
85. 70% ethanol (50 mL) (see Recipes)
86. Buffer KM (500 μL) (see Recipes)
87. 200 μM amino acid mixture (500 μL) (see Recipes)
88. 1× passive lysis buffer (5 mL) (see Recipes)

## Equipment

1. PIPETMAN P-10 (Gilson, catalog number: F144802)
2. PIPETMAN P-20 (Gilson, catalog number: F123600)
3. PIPETMAN P-200 (Gilson, catalog number: F123601)
4. PIPETMAN P-1000 (Gilson, catalog number: F123602)
5. PiptPAL single-channel pipette 1,000–10,000 μL (BMBio, catalog number: PAL-10 ml)
6. Pipet-Aid XP2 110 V, w/Charger (Drummond, catalog number: 4-040-501)
7. Optima MAX-TL ultracentrifuge (Beckman Coulter, catalog number: A95761)
8. TLA110 rotor (Beckman Coulter, catalog number: 366735)
9. Tube rack (13.0 mm, tubes) (Beckman Coulter, catalog number: 348122)
10. CO_2_ incubator (PHCbi, model: MCO-170AIC-PJ)
11. High-speed microcentrifuge (Hitachi, model: himac CF16RN)
12. Swing rotor (Hitachi, model: T4SS31) and 15TCX6S adaptor (Hitachi, catalog number: S307335A)
13. High-speed refrigerated microcentrifuge (TOMY, model: MX-307)
14. High-speed refrigerated microcentrifuge rotar rack (TOMY, model: AR015-24)
15. Analytical balance (SHIMADZU, model AP124W)
16. Slim stirrer same rotation control (AS ONE Corporation, model: 1-5940-02-22)
17. Semimicro stir bar (value) Ø3 mm × 6 mm football (AS ONE Corporation, model: 3-6659-03)
18. Qubit 2.0 fluorometer (Thermo Fisher Scientific)
19. Mini cooling dry bath incubator (Major Science, model: MC-0203) with mini dry bath blocks (Major Science, model: MD-MINI-B02)
20. Power Supply Power Station III (ATTO, model: WSE-3200)
21. Mini-Gel Slab Electrophoresis Tank (BIO CRAFT, model: BE-211G)
22. LABO SHAKER (BIO CRAFT, model: BC-740)
23. Odyssey CLx Imager (LI-COR, model: 9140)
24. ProFlex 3 × 32-well PCR system (Thermo Fisher Scientific, catalog number: 4484073)
25. Spectrophotometer (DeNovix, model: DS-11)
26. Milli-Q reference A+ system (MERCK, model: Z00QSVC01)
27. Mupid-exU submarine electrophoresis system (ADVANCE, model: EXU-1)
28. LED transilluminator (Gellex International ltd., model: LB-16)
29. Ultraviolet transilluminator (UVP, model: M-20)
30. MIXER uzusio (LMS, model: VTX-3000L)
31. MINI centrifuge (ALLSHENG, model: Mini-6KS)
32. NGS MagnaStand (YS-Model) 8 Ch × 0.2 mL PCR tube (FastGene, model: FG-SSMAG2)
33. Microchip Electrophoresis System for DNA/RNA Analysis MultiNA (SHIMADZU, model: MCE-202), equipped with MICROCHIP, TYPE WE-C (SHIMADZU, model: 292-36010-41)
34. GloMax Navigator System with Dual Injectors (Promega, model: GM2010)

## Software

1. Image Studio (LI-COR, ver. 5.2)
2. MultiNA Control Software (SHIMADZU, ver. 1.14.0)
3. MultiNA Viewer (SHIMADZU, ver. 1.14.0)
4. GloMax Navigator Software (Promega, ver. 3.1.0)
5. Excel (Microsoft, ver. 16.66.1)

## Procedure

- **Preparation of ribosome-depleted RRL**
  1. Load 1 mL of RRL into a 1.5 mL microcentrifuge tube, place into an 11 mm Delrin tube adapter, and ultracentrifuge at 240,000× *g* for 2 h 15 min at 4 °C using an Optima MAX-TL ultracentrifuge with a TLA110 rotor.
*Note: Keep the sample on ice as much as possible in steps 1–2. Handling the sample in a cold room should be an option.*
  2. Collect 900 μL of the supernatant, transfer to a 1.5 mL DNA LoBind tube, flash freeze with liquid nitrogen, and store at -80 °C. See Video 1 for details on the RRL supernatant collection.
*Notes:*
    - *Avoid touching the precipitate that contains the ribosomes.*
    - *Consider dividing the ribosome-depleted RRL into aliquots in several tubes before the flash freezing to avoid repeated freezethaw cycles.*
      **Video 1.**
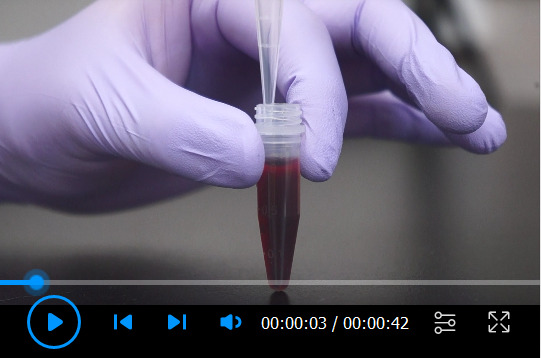
Collection of the RRL supernatant
- **Purification of ribosomes from HEK293 cells**
  1. Seed 5 × 10^6^ HEK293T (naïve or *METTL18* KO) cells in 10 mL of DMEM with 10% FBS (see Recipes) into a 10 cm dish and incubate overnight in a humidified incubator with 5% CO_2_ at 37 °C. Prepare 10 dishes.
*Note: The cell culture typically reaches 70%–80% confluency.*
  2. Aspirate the medium from a dish and add 5 mL of ice-cold PBS.
  3. Aspirate the PBS immediately and add 1 mL of ice-cold PBS.
  4. Repeat steps 2–3 for the other nine dishes.
  5. Resuspend cells in the dishes by pipetting, transfer to a 15 mL tube, and centrifuge at 500× *g* for 3 min at 4 °C, using a refrigerated centrifuge with a swing rotor.
  6. Discard the supernatant and add 1 mL of ice-cold PBS.
  7. Resuspend cells by pipetting, transfer to a 1.5 mL DNA LoBind tube, and centrifuge at 500× *g* for 3 min at 4 °C, using a refrigerated centrifuge with a fixed angle rotor.
*Note: Weigh the 1.5 mL tube before the cell transfer for the next step.*
  8. Discard the supernatant and weigh the tube with the cell pellet by the scale.
*Notes:*
    - *From this step, handle the sample on ice or at 4 °C.*
    - *We typically have a ~300 mg cell pellet.*
  9. Resuspend the cell pellet in the same volume of buffer R (see Recipes) (e.g., 300 μL of buffer R to 300 mg of cell pellet) and incubate for 15 min on ice.
*Note: Buffer R is a low-stringency buffer. Thus, the isolated ribosome may contain the associated factors.*
  10. Vortex the mixture for 30 s and centrifuge at 16,000× *g* for 10 min at 4 °C, using a refrigerated centrifuge with a fixed angle rotor.
  11. Collect the supernatant in a 1.5 mL DNA LoBind tube and mix well. Keep 10 μL of the supernatant in another 1.5 mL DNA LoBind tube for Coomassie Brilliant Blue staining.
  12. Load 300 μL of the supernatant at the bottom of the 3.2 mL polycarbonate tube and then underlay 1 mL of sucrose cushion solution (see Recipes) slowly using a PIPETMAN P-1000 with a long 1 mL tip. See Video 2 for details on the underlaying of sucrose cushion solution.
*Notes:*
    - *The cell lysate should float on top of the sucrose cushion solution. The interface between the cell lysate and sucrose cushion solution should be clearly visible. If the two solutions are mixed, the ribosome pellet may contain increased contaminants.*
    - *Instead of PIPETMAN P-1000 with a long 1 mL tip, a cannula or equivalent can be used.*
    **Video 2.**
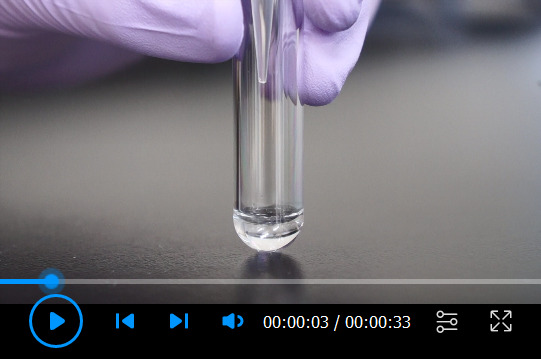
Laying sucrose cushion solution under the cell lysate
  13. Ultracentrifuge the tube at 240,000× *g* for 2 h 15 min at 4 °C using an Optima MAX-TL with a TLA110 rotor.
*Note: Mark the outside edge of the 3.2 mL polycarbonate tube to indicate the side where the ribosome pellet should be located.*
  14. Discard the supernatant by removing thoroughly with a pipette and slowly add 100 μL of buffer R2 (see Recipes) to the bottom of the tube, avoiding disrupting the pellet.
*Note: A glassy ribosome pellet should be visible on the outside edge of the tube bottom.*
  15. Discard the supernatant immediately and add 30 μL of buffer R2.
  16. Place the tube on a tube rack, add a stir bar to the tube, and then resuspend the solution at 4 °C for 15 min on the magnetic stirrer. See Video 3 for details.
    **Video 3.**
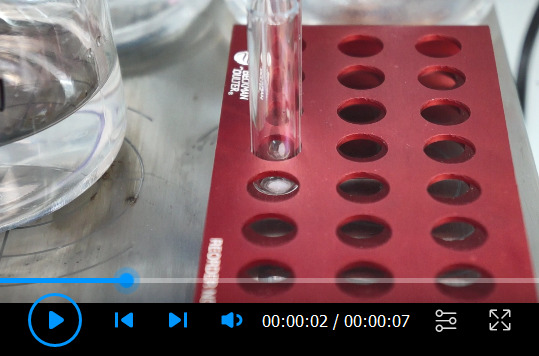
Resuspension of the pelleted ribosomes with a stir bar and a magnetic stirrer
  17. Transfer the solution to a 1.5 mL DNA LoBind tube. Keep 1 μL of the solution in another 1.5 mL DNA LoBind tube for Coomassie Brilliant Blue staining.
*Note: If the pellet does not dissolve completely, stir additionally for 15 min.*
  18. Determine the RNA concentration by Qubit RNA BR Assay kit and then determine the ribosome concentration as described below.
    - Prepare 200 μL of Qubit working solution for each standard and sample by diluting Qubit RNA BR reagent 200-fold with Qubit RNA BR buffer, according to the following table below. When you have one sample to test, prepare [2 (standards) + 1 (sample) + 1] × 200 μL solution.
      **Table:** 

Reagent	Final concentration	Amount
Qubit RNA BR buffer	n/a	800 μL
Qubit RNA BR reagent	n/a	4 μL
    - Prepare the assay tubes (use 0.5 mL PCR tubes accompanied with Qubit RNA BR Assay kit) according to the following table.
      Standards
      **Table:** 

Reagent	Final concentration	Amount
Qubit working solution	n/a	190 μL
20× Standard 1 or 2 (accompanied with Qubit RNA BR Assay kit)	1×	10 μL
      Samples
      **Table:** 

Reagent	Final concentration	Amount
Qubit working solution	n/a	199 μL
Ribosome solution	n/a	1 μL
    - Vortex standards and samples for 2–3 s and incubate at room temperature for 2 min.
    - Turn on Qubit 2.0 fluorometer and select *RNA* and then *RNA Broad Range Assay*. Calibrate with the standards and then read the sample.
    - Select *Read Next Sample*, followed by *Calculate Stock Conc*. and then *1 μL* to calculate the concentration of RNA in the ribosome solution.
*Note: We typically have 1,000–2,000 ng/μL RNA in the ribosome solution.*
    - Convert the RNA concentration to the ribosome concentration. Given the size of rRNAs (18S rRNA, 1.9 kb; 28S rRNA, 5 kb), the formula (number of nucleotides × 320.5) provides a total rRNA molecular weight of 6.9 × 10^3^ × 320.5 = 2.2 × 10^6^. Thus, the molar concentration should be determined as X [ng/μL]/(2.2 × 10^6^). For example, for a ribosome solution with 1,500 ng/μL RNA, the ribosome concentration should be 1,500 [ng/μL]/(2.2 × 10^6^) = 0.68 μM.
  19. Dilute the ribosome solution to 226 nM with buffer R2.
  20. Flash freeze the purified ribosome with liquid nitrogen and store at -80 °C.
*Note: Consider dividing the ribosome solution into aliquots in several tubes before the flash freeze to avoid repeated freezethaw cycles.*
  21. Determine the protein composition in the ribosome solution by Coomassie Brilliant Blue staining.
    - Mix the HEK293T cell lysate (from step 11) and ribosome solution (from step 17) with 2× Laemmli sample buffer (see Recipes) in each 1.5 mL DNA LoBind tube according to the table below and denature at 95 °C for 10 min in a dry bath incubator.
      **Table:** 

Reagent	Cell lysate	Ribosome solution
Sample	10 μL	1 μL
Buffer R2	n/a	9 μL
2× Laemmli sample buffer	10 μL	10 μL
    - Load 20 μL of each sample and 5 μL of BlueStar prestained protein ladder into a precast 5%–20% polyacrylamide gel in 1× SDS-PAGE running buffer (see Recipes) set up in the mini-gel electrophoresis tank. Perform electrophoresis at a constant 20 mA for 70 min at room temperature with a power supply.
    - Fix the gel with 15 mL of gel fixation buffer (see Recipes) for 30 min on a rotary shaker at room temperature and briefly wash the gel twice with Milli-Q water.
    - Stain the gel with 15 mL of GelCode Blue Safe protein stain for 60 min on a rotary shaker and wash the gel with Milli-Q water.
    - Destain the gel with Milli-Q water and paper towels until the background signals are reduced.
*Note: This typically takes 3 h to overnight.*
    - Visualize the gel on an Odyssey CLx imager and Image Studio with 700 nm channels with the focus at 0.5 mm, which corresponds to the center of the 1 mm thick gel. See Figure 1 for representative protein staining results.
    **Figure 1.**
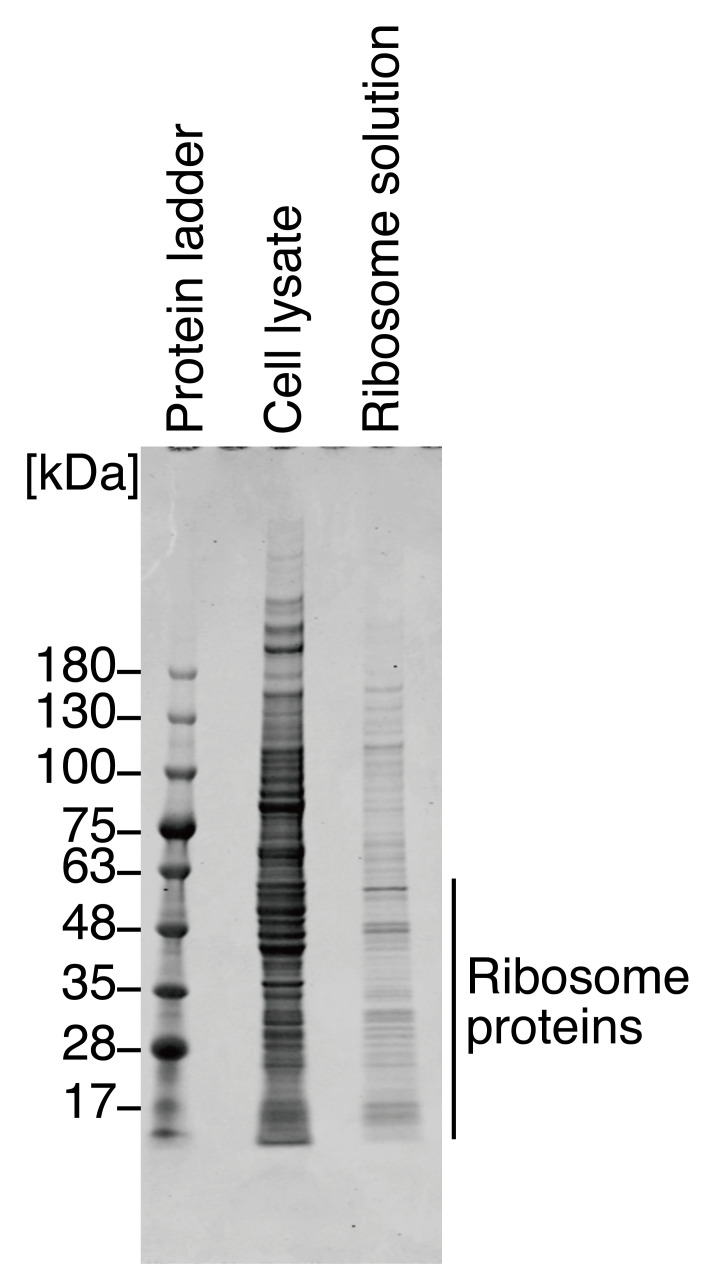
Coomassie Brilliant Blue staining for proteins in ribosome solution. Representative gel image of Coomassie Brilliant Blue staining for proteins in HEK293T cell lysate (i.e., input) and in prepared ribosome solution. The staining pattern was similar to that in an earlier report (Penzo et al., 2016). We note that due to the low stringency of buffer R, the ribosome solution contained high-molecular-weight ribosome-interacting proteins in addition to ribosomal proteins.
- **Preparation of reporter mRNAs**
  1. Prepare the PCR mixture in a 0.2 mL PCR tube as described below to amplify the DNA fragments for the template of in vitro transcription of Rluc-Y39×-Fluc and Rluc-Y0×-Fluc RNAs (100 μL reaction).
    **Table:** 

Reagent	Final concentration	Amount
1 ng/μL plasmid (psiCHECK2-Y0× or psiCHECK2-Y39×)	0.01 ng/μL	1 μL
5 μM Primer 1	0.2 μM	4 μL
5 μM Primer 2	0.2 μM	4 μL
PrimeSTAR Max Premix (2×)	1×	50 μL
RNase-free water	n/a	41 μL
  2. Perform the PCR amplification in a thermal cycler with the program described below.
    **Table:** 

Temperature	Time	Cycles
98 °C	10 s	1×
98 °C	10 s	30×
55 °C	5 s	
72 °C	35 s	
72 °C	1 min	1×
4 °C	∞	1×
  3. Mix the PCR with 10× loading buffer, load it onto 1% agarose gel (see Recipes) (with 1 kb DNA ladder on a lane), and separate the DNA fragment by electrophoresis with TAE (see Recipes).
  4. Stain the gel with GreenView nucleic acid gel stain in TAE and visualize the nucleic acid using a blue light LED transilluminator. See Figure 2 for the representative results for the Rluc-Y39×-Fluc reporter template.
  5. Gel-purify the appropriate size of DNA fragments (~2.8 kbp) with NucleoSpin Gel and PCR Clean-up according to the manufacturer’s instructions.
  6. Measure the concentration of template DNA using a DS-11 spectrophotometer.
  7. Prepare the in vitro transcription reaction using the T7-Scribe Standard RNA IVT kit in 0.2 mL PCR tubes as described below (20 μL reaction).
    **Table:** 

Reagent	Final concentration	Amount
Purified DNA template (Rluc-Y39×-Fluc or Rluc-Y0×-Fluc)	1 μg	X μL
10× T7-Scribe transcription buffer	1×	2 μL
100 mM ATP	7.5 mM	1.5 μL
100 mM CTP	7.5 mM	1.5 μL
100 mM UTP	7.5 mM	1.5 μL
100 mM GTP	7.5 mM	1.5 μL
100 mM DTT	10 mM	2 μL
40 U/μL ScriptGuard RNase inhibitor	1 U/μL	0.5 μL
T7-Scribe enzyme solution	n/a	2 μL
RNase-free water	n/a	7.5 - X μL
  8. Incubate at 37 °C for 2 h in a thermal cycler.
*Note: Extension of the incubation time to 4–6 h may increase the yield of RNA.*
  9. Add 1 μL of DNase I (a component of the T7-Scribe Standard RNA IVT kit) and incubate at 37 °C for 2 h in a thermal cycler.
*Note: RNA may be stored at -20 °C before purification.*
  10. Purify the RNA as described below.
    - Mix the bottle of the Agencourt RNAClean XP thoroughly to achieve homogeneous resuspension.
    - Add 1.8× volume of bead solution to the reaction, mix well by pipetting 10 times or vortexing for 30 s, and centrifuge the tube briefly.
    - Incubate at room temperature for 15 min and place the tube on the magnetic stand for 5 min to separate the beads from the solution.
    - Discard the cleared supernatant.
    - Add 200 μL of 70% ethanol (see Recipes) into the tube and incubate for 30 s at room temperature.
*Note: Keep the tube standing on the magnetic stand during steps e–f.*
    - Discard the cleared supernatant.
    - Repeat steps e–f twice (for a total of three washes).
    - Centrifuge the tube briefly to collect the remaining 70% ethanol at the bottom of the tube, keep the tube on the magnetic stand for 1 min, and discard the cleared supernatant.
    - Open the lid of the tube and allow the beads to dry for 5 min at room temperature.
*Note: Beads may be cracked after completely drying.*
*Caution: Overdrying the beads may result in inefficient recovery of RNA.*
    - Add 20 μL of RNase-free water, resuspend the beads by pipetting, and incubate for 2 min at room temperature.
    - Place the tube on the magnetic stand for 2 min at room temperature and then transfer the cleared supernatant to a new 1.5 mL DNA LoBind tube.
*Note: The RNA may be stored at -20 °C or -80 °C.*
    - Measure the concentration of the RNA using a DS-11 spectrophotometer.
*Note: We typically have ~1 μg/μL RNA solution.*
  11. Prepare the following solution in 0.2 mL PCR tubes (33.5 μL reaction volume).
    **Table:** 

Reagent	Final concentration	Amount
Purified RNA	20–30 μg	X μL
RNase-free water	n/a	33.5 - X μL
*Note: Keep the remaining RNA for application to the fragment analyzer.*
  12. Incubate at 65 °C for 5 min in a thermal cycler and then immediately place on ice.
  13. Prepare the capping reaction using the ScriptCap m^7^G Capping System and ScriptCap 2′-*O*-Methyltransferase kit in the 0.2 mL PCR tubes as described below (50 μL reaction).
    **Table:** 

Reagent	Final concentration	Amount
Denatured RNA	20–30 μg	33.5 μL
10× ScriptCap capping buffer	1×	5 μL
10 mM GTP	1 mM	5 μL
20 mM SAM	0.5 mM	1.25 μL
40 U/μL ScriptGuard RNase inhibitor	1 mM	1.25 μL
100 U/μL ScriptCap 2′-*O*-methyltransferase	4 U/μL	2 μL
10 U/μL ScriptCap capping enzyme	0.4 U/μL	2 μL
  14. Incubate at 37 °C for 30 min in a thermal cycler.
  15. Prepare the poly(A) tailing reaction using the A-Plus Poly(A) Polymerase Tailing Kit in 0.2 mL PCR tubes as described below (66 μL reaction volume).
    **Table:** 

Reagent	Final concentration	Amount
Capping reaction	n/a	50 μL
40 U/μL ScriptGuard RNase inhibitor	0.18 U/μL	0.3 μL
10× A-Plus poly(A) tailing buffer	1×	6.6 μL
20 mM ATP	2 mM	6.6 μL
4 U/μL A-Plus poly(A) polymerase	0.15 U/μL	2.5 μL
  16. Incubate at 37 °C for 30 min in a thermal cycler.
  17. Add 2.5 μL of 0.5 M EDTA to stop the poly(A) tailing reaction (to make ~18 mM EDTA at final concentration).
*Note: The poly(A) tailing reaction may be stored at -20 °C before purification.*
  18. Purify the RNA as described in step 10 above.
*Note: Purified RNA may be stored at -20 °C or -80 °C before use.*
  19. Assess the purity of the RNA with the fragment analyzer MultiNA.
*Note: Instead of MultiNA, Bioanalyzer, TapeStation, or electrophoresis with denaturing TBE-agarose gel can be used.*
    - Bring separation buffer (a reagent of the RNA 1000 kit), marker solution (a reagent of the RNA 1000 kit), SYBR Green stock solution, and RNA 6000 ladder to room temperature.
    - Dilute SYBR Green stock solution 100-fold with TE.
    - Dilute RNA 6000 Ladder 6-fold with TE.
    - Dilute poly(A)-tailed reporter RNA (from step 18) and non-poly(A)-tailed RNA (from steps 10–11) with RNase-free to 25–250 ng/μL.
    - Prepare the required volume of buffer solution for MultiNA into a screw cap tube, 5 mL, as described below.
*Note: The required volume depends on the sample number. The following table shows an example.*
      **Table:** 

Reagent	Final concentration	Amount
1/100 diluted SYBR Green	n/a	1 μL
Formamide	n/a	80 μL
Separation buffer (a reagent of the RNA 1000 kit)	n/a	319 μL
    - Prepare the sample and RNA 6000 ladder in 0.2 mL PCR tubes for the MultiNA run as described below.
      **Table:** 

Reagent	Final concentration	Amount
1/6 RNA 6000 ladder, 1/10 poly(A)-tailed reporter RNA, or 1/10 non-poly(A)-tailed reporter RNA	n/a	3 μL
RNA marker solution (a reagent of the RNA 1000 kit)	n/a	3 μL
    - Incubate at 65 °C for 5 min in a thermal cycler and immediately place on ice.
    - Set the 0.2-mL PCR tubes from step f and the 5 mL tube from step e (leaving the lid off) on MultiNA and start the run with MultiNA Control Software. See Figure 3 for the representative results for the Rluc-Y39×-Fluc reporter transcript.
    - Calculate the molar concentration of reporter mRNA as described below.
      i. Take the ng/μL concentration by the result of the MultiNA. For example, since the concentration of Rluc-Y39×-Fluc with poly(A) tail in Figure 3 was 6.0 ng/μL, the stock solution should be 6.0 ng/μL × 10 (dilution factor from the stock) = 60 ng/μL.
      ii. Estimate the poly(A) length by the result of MultiNA. For example, the poly(A) length of Rluc-Y39×-Fluc with a poly(A) tail in Figure 3 was ~100 nt.
      iii. Calculate the molecular weight of reporter mRNA. Given that the non-poly(A)-tailed Rluc-Y39×-Fluc should be 2,787 nt, the poly(A)-tailed Rluc-Y39×-Fluc should be ~2,887 nt. Thus, the estimated molecular weight should be 2,887 × 320.5 = 0.93 × 10^6^. For example, the molar concentration of poly(A)-tailed Rluc-Y39×-Fluc reporter in Figure 3 should be 60 [ng/μL]/(0.93 × 10^6^) = 65 nM.
  20. Dilute the reporter mRNA to 11 nM with RNase-free water.
    **Figure 2.**
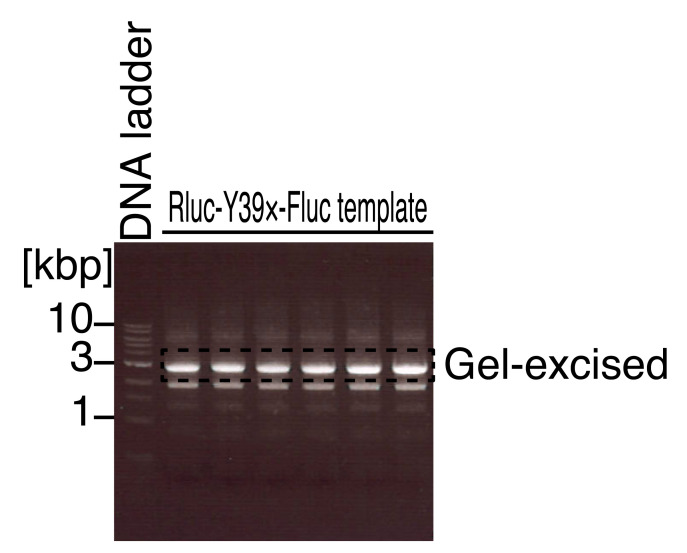
Agarose gel electrophoresis for the PCR-amplified DNA fragments for in vitro transcription templates. Representative gel image of the 1 kb DNA ladder and the PCR product for the in vitro transcription template of the Rluc-Y39×-Fluc reporter. The area indicated by the dashed line was gel excised. Note that this image was visualized by a UV transilluminator, which should be avoided for the actual gel-excision step.
    **Figure 3.**
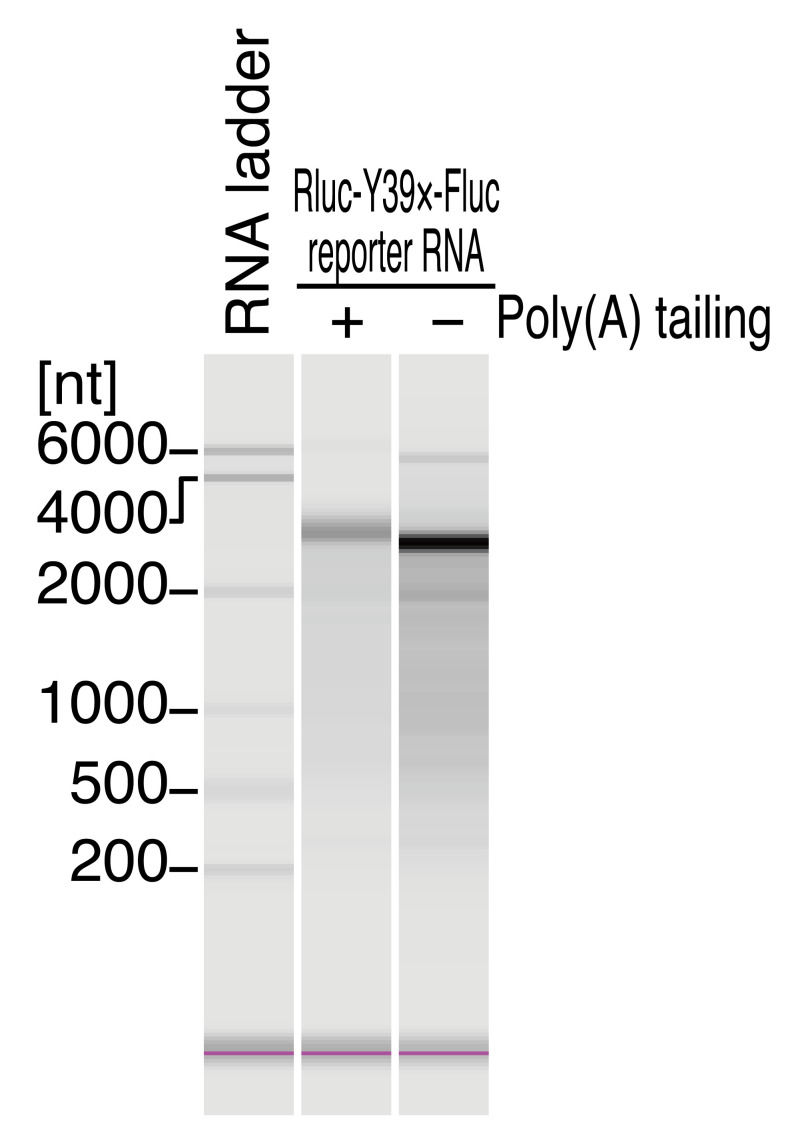
Quality check of the reporter transcript by fragment analyzer. Representative electropherograms of RNA 6000 ladder and Rluc-Y39×-Fluc reporter RNA with and without poly(A) tail. MultiNA Viewer software automatically assesses the size and concentration of RNA. The poly(A) tail length was estimated to be ~100 nt based on the length difference. Additionally, the RNA concentrations were determined as follows: Rluc-Y39×-Fluc with poly(A) tail, 6.0 ng/μL; Rluc-Y39×-Fluc without poly(A) tail, 23.3 ng/μL. We note that the concentration of individual mRNA measured by MultiNA may be lower than the total RNA concentration predicted by the DS-11 spectrophotometer.
- **Translation reaction**
  1. Prepare the in vitro translation reaction in a 1.5 mL DNA LoBind tube as described below (120 μL reaction).
    **Table:** 

Reagent	Final concentration	Amount
Ribosome-depleted RRL	n/a	60 μL
226 nM purified ribosome (from naïve or *METTL18* KO cells)	22.6 nM	12 μL
11 nM reporter mRNA (Rluc-Y39×-Fluc or Rluc-Y0×-Fluc)	1.1 nM	12 μL
Buffer KM (see Recipes)	KCl: 75 mM MgCl_2_: 0.75 mM	12 μL
200 μM amino acid mixture (see Recipes)	20 μM	12 μL
40 U/μL RNase inhibitor (TaKaRa)	0.8 U/μL	2.4 μL
RNase-free water	n/a	9.6 μL
*Note: RRL includes an energy regeneration system (phosphocreatine and phosphocreatine kinase), hemin, tRNA mixture, etc., which are generally required for in vitro translation. Thus, further supplementation of those materials is not necessary.*
  2. Start the reaction at 25 °C, take a 10 μL aliquot from the reaction every 5 min (for 50 min total, i.e., total 11 timepoints, including time 0 min) into 1.5 mL DNA LoBind tubes, immediately mix with 20 μL of 1× passive lysis buffer (see Recipes) to quench the reaction, and keep the mixture on ice until the luminescence measurement.
  3. Measure the Rluc and Fluc luminescence by the Dual-Luciferase Reporter Assay System in Glomax.
    - Prepare Luciferase assay reagent II (LAR II) working solution as described below (for 44 + 1 samples).
      **Table:** 

Reagent	Final concentration	Amount
Luciferase assay substrate (lyophilized product)	n/a	1 vial
Luciferase assay buffer II	n/a	10 mL
*Notes:*
*i. Typically, the assay requires (number of samples +1) × 50 + 500 μL.*
*ii. The remaining LAR II can be stored at −80 °C for a month.*
    - Prepare Stop & Glo working solution as described below.
      **Table:** 

Reagent	Final concentration	Amount
50× Stop & Glo substrate	1×	55 μL
Stop & Glo buffer	n/a	2,695 μL
*Note: Typically, the assay requires (number of samples +1) × 50 + 500 μL.*
    - Wash both injectors of the GloMax Navigator System with Milli-Q water, 70% ethanol, Milli-Q water, and air, according to the manufacturer’s instructions.
    - Prime injectors with LAR II and Stop & Glo working solutions according to the manufacturer’s instructions.
    - Program the GloMax Navigator Software to perform a two-second premeasurement delay, followed by a 10-second measurement period for each assay, and to dispense 50 μL of LAR II and Stop & Glo working solutions per sample.
    - Transfer 20 μL of the reaction mixture (prepared in step 2 above) to each well of a white 96-well plate.
    - Place the white 96-well plate in the GloMax Navigator System and initiate the assay.
    - Export the luminescence data from the GloMax Navigator Software into a csv file. See the representative data of “GloMax_rawdata_1.csv” in Supplemental material.

## Data analysis

1. Load the csv file into Excel software and analyze the data for the following steps. For the representative data analysis, see “Slope.xlsx” in Supplemental material. See Figure 4 for representative data analysis results.

- Draw the scatter plots of Rluc and Fluc luminescence along the time.
- Determine the linear part of the signal raise and calculate the slope (Slope_Rluc_ and Slope_Fluc_).
*Note: In this analysis, we used luminescence values at 25–45 min. The timepoint that provides a linear increase in the signal may depend on the experimental conditions.*
- Calculate Slope_Fluc_/Slope_Rluc_ and then relative Slope_Fluc_/Slope_Rluc_ (i.e., normalize the Slope_Fluc_/Slope_Rluc_ of *METTL18* KO ribosomes by that of naïve ribosomes).
  **Figure 4.**
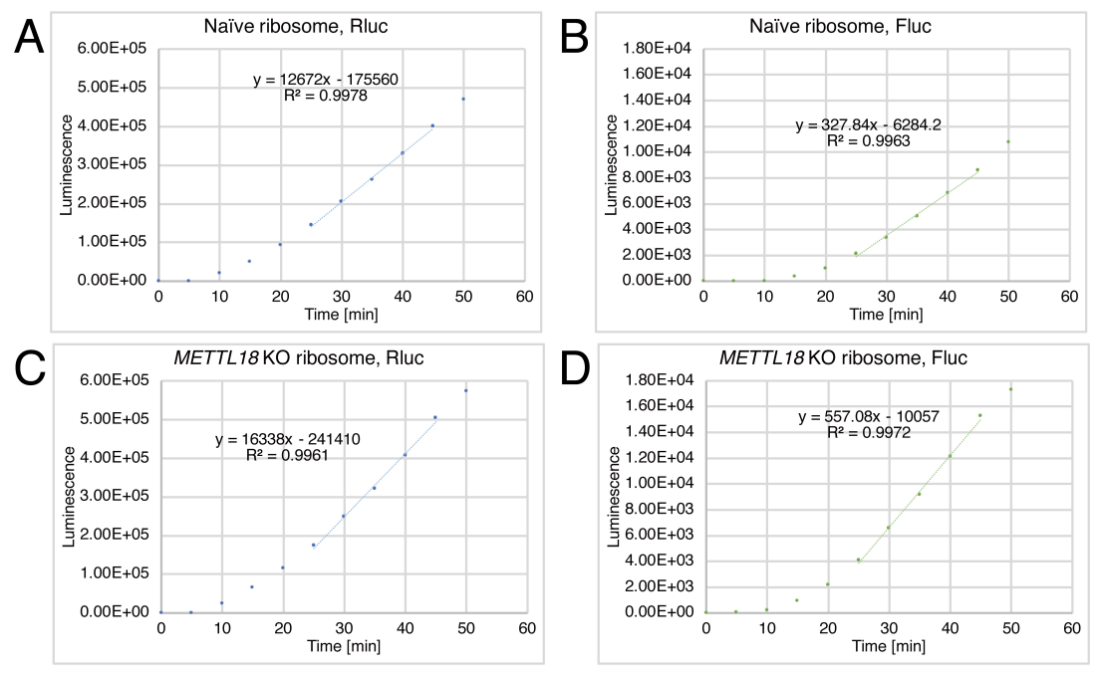
Representative data of the hybrid translation. Rluc and Fluc luminescence from the Rluc-Y39×-Fluc reporter over incubation time. Data for naïve ribosomes (A and B) and for *METTL18* KO ribosomes (C and D) are shown. Based on the results, the Slope_Fluc_/Slope_Rluc_ values for naïve ribosomes and *METTL18* KO ribosomes were determined to be 0.0259 and 0.0341, respectively. Thus, the relative Slope_Fluc_/Slope_Rluc_ for *METTL18* KO ribosomes was 1.32, suggesting increased processivity of elongation along Y39×.

## Notes

**Figure 5.**
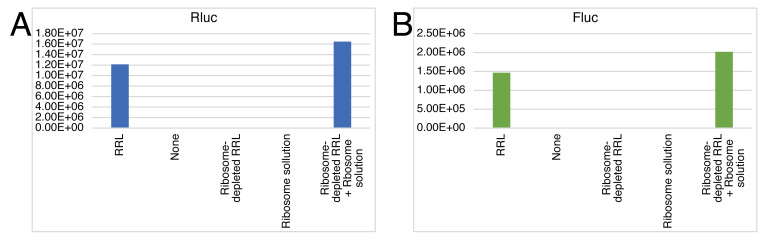
Control experiments for checking the quality of materials used in the hybrid translation. Rluc (A) and Fluc (B) luminescence from the Rluc-Y0×-Fluc reporter under the indicated conditions after 60 min of incubation. Data for naïve ribosomes are shown.

To test whether individual ribosome-depleted RRL and purified ribosomes have no translation activity on their own, small-scale reactions (10 μL) omitting each factor should be conducted prior to the experiments. See Figure 5 for the representative result, “GloMax_rawdata_2.csv” in Supplemental material for the raw data, and “Material_check.xlsx” in Supplemental material for the analysis.

## Recipes

1. **DMEM supplemented with FBS (500 mL)**
  **Table:** 

Reagent	Final concentration	Amount
DMEM	n/a	500 mL
FBS	10%	50 mL
*Note: Keep at 4 °C.*
2. **1 M KOAc (5 mL)**
  **Table:** 

Reagent	Final concentration	Amount
KOAc	1 M	0.49 g
RNase-free water	n/a	up to 5 mL
*Note: Keep at room temperature.*
3. **1 M MgOAc_2_ (1 mL)**
  **Table:** 

Reagent	Final concentration	Amount
MgOAc_2_·4H_2_O	1 M	0.214 g
RNase-free water	n/a	up to 1 mL
*Note: Keep at room temperature.*
4. **1 M DTT (5 mL)**
  **Table:** 

Reagent	Final concentration	Amount
DTT	1 M	0.771 g
RNase-free water	n/a	up to 5 mL
*Note: Store at -20 °C.*
5. **Buffer R (10 mL)**
  **Table:** 

Reagent	Final concentration	Amount
1 M HEPES-KOH pH 7.5	10 mM	100 μL
1 M KOAc	10 mM	100 μL
1 M MgOAc_2_	1 mM	10 μL
1 M DTT	1 mM	10 μL
RNase-free water	n/a	9,780 μL
*Note: Prepare before use and keep on ice.*
6. **Sucrose cushion solution (10 mL for eight samples)**
  **Table:** 

Reagent	Final concentration	Amount
Sucrose	1 M	3.4 g (corresponding to 2.2 mL)
1 M HEPES-KOH pH 7.5	10 mM	100 μL
1 M KOAc	10 mM	100 μL
1 M MgOAc_2_	1 mM	10 μL
1 M DTT	1 mM	10 μL
RNase-free water	n/a	7,580 μL
*Note: Prepare before use and keep on ice.*
7. **Buffer R2 (5 mL)**
  **Table:** 

Reagent	Final concentration	Amount
1 M HEPES-KOH pH 7.5	20 mM	100 μL
5 M NaCl	10 mM	10 μL
2 M KCl	25 mM	62.5 μL
1 M MgCl_2_	1.1 mM	5.5 μL
14.3 M 2-mercaptoethanol	7.7 mM	2.7 μL
RNase-free water	n/a	4819.3 μL
*Note: Prepare before use and keep on ice.*
8. **2× Laemmli sample buffer (1 mL)**
  **Table:** 

Reagent	Final concentration	Amount
1 M Tris-HCl pH 6.8	125 mM	125 μL
100% Glycerol	20%	200 μL
10% SDS solution	4%	400 μL
Bromophenol Blue	0.004%	0.04 mg
RNase-free water	n/a	175 μL
14.3 M 2-mercaptoethanol	10%	100 μL
*Notes:*
  - *Keep at room temperature (without 2-mercaptoethanol).*
  - *Add 2-mercaptoethanol before use.*
9. **10× SDS-PAGE running buffer (1 L)**
  **Table:** 

Reagent	Final concentration	Amount
Tris(hydroxymethyl)aminomethane	0.25 M	30.2 g
Glycine	1.92 M	144 g
Sodium lauryl sulfate	1%	10 g
Milli-Q water	n/a	up to 1 L
*Note: Keep at room temperature.*
10. **1× SDS-PAGE running buffer (1 L)**
  **Table:** 

Reagent	Final concentration	Amount
10× Running buffer	1×	100 mL
Milli-Q water	n/a	900 mL
*Note: Keep at room temperature.*
11. **Gel fixation buffer (100 mL)**
  **Table:** 

Reagent	Final concentration	Amount
Methanol	50%	50 mL
Acetic acid	7%	7 mL
Milli-Q water	n/a	43 mL
*Note: Keep at room temperature.*
12. **50× TAE (1 L)**
  **Table:** 

Reagent	Final concentration	Amount
Tris(hydroxymethyl)aminomethane	2 M	242 g
Acetic acid	1 M	57.1 mL
0.5 M EDTA (pH 8.0)	50 mM	100 mL
Milli-Q water	n/a	up to 1 L
13. **1% agarose gel (100 mL)**
  **Table:** 

Reagent	Final concentration	Amount
Agarose	1%	1 g
50× TAE	1×	2 mL
Milli-Q water	n/a	97 mL
*Note: Dissolve agarose by heating with a microwave oven. Pour the reagent into the gasket and cool it until use.*
14. **70% ethanol (50 mL)**
  **Table:** 

Reagent	Final concentration	Amount
Ethanol	70%	35 mL
RNase-free water	n/a	15 mL
15. **Buffer KM (500 μL)**
  **Table:** 

Reagent	Final concentration	Amount
2 M KCl	750 mM	187 μL
1 M MgCl_2_	7.5 mM	3.7 μL
RNase-free water	n/a	309.3 μL
*Note: Prepare before use and keep on ice.*
16. **200 μM amino acid mixture (500 μL)**
  **Table:** 

Reagent	Final concentration	Amount
1 mM amino acid mixtures	200 μM	100 μL
RNase-free water	n/a	400 μL
*Note: Store at -80 °C.*
17. **1× passive lysis buffer (5 mL)**
  **Table:** 

Reagent	Final concentration	Amount
5× passive lysis buffer	1×	1 mL
RNase-free water	n/a	4 mL
*Note: Prepare before use.*

## Supplementary information

The following supporting information can be downloaded here:

1. GloMax_rawdata_1.csv
2. GloMax_rawdata_2.csv
3. Slope.xlsx
4. Material_check.xlsx

## References

[1] Abe T., Nagai R., Shimazaki S., Kondo S., Nishimura S., Sakaguchi Y., Suzuki T., Imataka H., Tomita K. and Takeuchi-Tomita N.(2020). In vitro yeast reconstituted translation system reveals function of eIF5A for synthesis of long polypeptide. J Biochem 167(5): 451-462. 32053170 10.1093/jb/mvaa022

[2] Alkalaeva E. Z., Pisarev A. V., Frolova L. Y., Kisselev L. L. and Pestova T. V.(2006). In vitro reconstitution of eukaryotic translation reveals cooperativity between release factors eRF1 and eRF3. Cell 125(6): 1125-1136. 16777602 10.1016/j.cell.2006.04.035

[3] Bergamini G., Preiss T. and Hentze M. W.(2000). Picornavirus IRESes and the poly(A) tail jointly promote cap-independent translation in a mammalian cell-free system. RNA 6(12): 1781-1790. 11142378 10.1017/s1355838200001679 PMC1370048

[4] Brödel A. K., Sonnabend A. and Kubick S.(2014). Cell-free protein expression based on extracts from CHO cells. Biotechnol Bioeng 111(1): 25-36. 24018795 10.1002/bit.25013

[5] Dieterich D. C., Hodas J. J., Gouzer G., Shadrin I. Y., Ngo J. T., Triller A., Tirrell D. A. and Schuman E. M.(2010). In situ visualization and dynamics of newly synthesized proteins in rat hippocampal neurons. Nat Neurosci 13(7): 897-905. 20543841 10.1038/nn.2580 PMC2920597

[6] Emmott E., Jovanovic M. and Slavov N.(2019). Ribosome stoichiometry: From form to function. Trends Biochem Sci 44(2): 95-109. 30473427 10.1016/j.tibs.2018.10.009 PMC6340777

[7] Erales J., Marchand V., Panthu B., Gillot S., Belin S., Ghayad S. E., Garcia M., Laforêts F., Marcel V., Baudin-Baillieu A., .(2017). Evidence for rRNA 2′-*O*-methylation plasticity: Control of intrinsic translational capabilities of human ribosomes. Proc Natl Acad Sci U S A 114(49): 12934-12939. 29158377 10.1073/pnas.1707674114 PMC5724255

[8] Ferretti M. B. and Karbstein K.(2019). Does functional specialization of ribosomes really exist? RNA 25(5): 521-538. 30733326 10.1261/rna.069823.118 PMC6467006

[9] Fritz S. E., Haque N. and Hogg J. R.(2018). Highly efficient in vitro translation of authentic affinity-purified messenger ribonucleoprotein complexes. RNA 24(7): 982-989. 29724884 10.1261/rna.065730.118 PMC6004058

[10] Genuth N. R. and Barna M.(2018). Heterogeneity and specialized functions of translation machinery: from genes to organisms. Nat Rev Genet 19(7): 431-452. 29725087 10.1038/s41576-018-0008-z PMC6813789

[11] Genuth N. R. and Barna M.(2018). The Discovery of Ribosome Heterogeneity and Its Implications for Gene Regulation and Organismal Life. Mol Cell 71(3): 364-374. 30075139 10.1016/j.molcel.2018.07.018 PMC6092941

[12] Gregorio N. E., Levine M. Z. and Oza J. P.(2019). A User’s Guide to Cell-Free Protein Synthesis. Methods Protoc 2(1). 10.3390/mps2010024 PMC6481089 31164605

[13] Guo H.(2018). Specialized ribosomes and the control of translation. Biochem Soc Trans 46(4): 855-869. 29986937 10.1042/BST20160426

[14] Hunt T. and Jackson R. J.(1974). The rabbit reticulocyte lysate as a system for studying mRNA. Hamatol Bluttransfus 14: 300-307. 4448420

[15] Ingolia N. T., Ghaemmaghami S., Newman J. R. and Weissman J. S.(2009). Genome-wide analysis *in vivo* of translation with nucleotide resolution using ribosome profiling. Science 324(5924): 218-223. 19213877 10.1126/science.1168978 PMC2746483

[16] Iwasaki S. and Ingolia N. T.(2017). The growing toolbox for protein synthesis studies. Trends Biochem Sci 42(8): 612-624. 28566214 10.1016/j.tibs.2017.05.004 PMC5533619

[17] Jackson R. J. and Hunt T.(1983). Preparation and use of nuclease-treated rabbit reticulocyte lysates for the translation of eukaryotic messenger RNA. Methods Enzymol 96: 50-74. 6656641 10.1016/s0076-6879(83)96008-1

[18] Kerr I. M., Cohen N. and Work T. S.(1966). Factors controlling amino acid incorporation by ribosomes from krebs II mouse ascites-tumour cells. Biochem J 98(3): 826-835. 4287844 10.1042/bj0980826 PMC1264925

[19] Kisly I., Remme J. and Tamm T.(2018). Ribosomal protein eL24, involved in two intersubunit bridges, stimulates translation initiation and elongation. Nucleic Acids Res 47(1): 406-420. 10.1093/nar/gky1083 PMC6326817 30407570

[20] Kisly I., Kattel C., Remme J. and Tamm T.(2021). Luciferase-based reporter system for in vitro evaluation of elongation rate and processivity of ribosomes. Nucleic Acids Res 49(10): e59. 33684199 10.1093/nar/gkab121 PMC8191769

[21] Machida K., Shigeta T., Yamamoto Y., Ito T., Svitkin Y., Sonenberg N. and Imataka H.(2018). Dynamic interaction of poly(A)-binding protein with the ribosome. Sci Rep 8(1): 17435. 30487538 10.1038/s41598-018-35753-1 PMC6261967

[22] Małecki J. M., Odonohue M.-F., Kim Y., Jakobsson M. E., Gessa L., Pinto R., Wu J., Davydova E., Moen A., Olsen J. V., .(2021). Human METTL18 is a histidine-specific methyltransferase that targets RPL3 and affects ribosome biogenesis and function. Nucleic Acids Res 49(6): 3185-3203. 33693809 10.1093/nar/gkab088 PMC8034639

[23] Mathews M. B. and Korner A.(1970). Mammalian Cell-Free Protein Synthesis Directed by Viral Ribonucleic Acid. Eur J Biochem 17(2): 328-338. 5500401 10.1111/j.1432-1033.1970.tb01170.x

[24] Matsuura-Suzuki E., Shimazu T., Takahashi M., Kotoshiba K., Suzuki T., Kashiwagi K., Sohtome Y., Akakabe M., Sodeoka M., Dohmae N., .(2022). METTL18-mediated histidine methylation of RPL3 modulates translation elongation for proteostasis maintenance. Elife 11: e72780. 35674491 10.7554/eLife.72780 PMC9177149

[25] Mikami S., Masutani M., Sonenberg N., Yokoyama S. and Imataka H.(2006). An efficient mammalian cell-free translation system supplemented with translation factors. Protein Expr Purif 46(2): 348-357. 16289705 10.1016/j.pep.2005.09.021

[26] Molla A., Paul A. V. and Wimmer E.(1991). Cell-free, de novo synthesis of poliovirus. Science 254(5038): 1647-1651. 1661029 10.1126/science.1661029

[27] Morisaki T., Lyon K., DeLuca K. F., DeLuca J. G., English B. P., Zhang Z., Lavis L. D., Grimm J. B., Viswanathan S., Looger L. L., .(2016). Real-time quantification of single RNA translation dynamics in living cells. Science 352(6292): 1425-1429. 27313040 10.1126/science.aaf0899

[28] Panthu B., Décimo D., Balvay L. and Ohlmann T.(2015). In vitro translation in a hybrid cell free lysate with exogenous cellular ribosomes. Biochem J 467(3): 387-398. 25628018 10.1042/BJ20141498

[29] Pelham H. R. B. and Jackson R. J.(1976). An efficient mRNA-dependent translation system from reticulocyte lysates. Eur J Biochem 67(1): 247-256. 823012 10.1111/j.1432-1033.1976.tb10656.x

[30] Penzo M., Rocchi L., Brugiere S., Carnicelli D., Onofrillo C., Couté Y., Brigotti M. and Montanaro L.(2015). Human ribosomes from cells with reduced dyskerin levels are intrinsically altered in translation. FASEB J 29(8): 3472-3482. 25934701 10.1096/fj.15-270991

[31] Penzo M., Carnicelli D., Montanaro L. and Brigotti M.(2016). A reconstituted cell-free assay for the evaluation of the intrinsic activity of purified human ribosomes. Nat Protoc 11(7): 1309-1325. 27336708 10.1038/nprot.2016.072

[32] Pestova T. V., Borukhov S. I. and Hellen C. U.(1998). Eukaryotic ribosomes require initiation factors 1 and 1A to locate initiation codons. Nature 394(6696): 854-859. 9732867 10.1038/29703

[33] Pestova T. V. and Hellen C. U. T.(2003). Translation elongation after assembly of ribosomes on the Cricket paralysis virus internal ribosomal entry site without initiation factors or initiator tRNA. Genes Dev 17(2): 181-186. 12533507 10.1101/gad.1040803 PMC195975

[34] Pisarev A. V., Hellen C. U. T. and Pestova T. V.(2007). Recycling of eukaryotic posttermination ribosomal complexes. Cell 131(2): 286-299. 17956730 10.1016/j.cell.2007.08.041 PMC2651563

[35] Rakotondrafara A. M. and Hentze M. W.(2011). An efficient factor-depleted mammalian in vitro translation system. Nat Protoc 6(5): 563-571. 21527914 10.1038/nprot.2011.314

[36] Schwanhäusser B., Gossen M., Dittmar G. and Selbach M.(2009). Global analysis of cellular protein translation by pulsed SILAC. Proteomics 9(1): 205-209. 19053139 10.1002/pmic.200800275

[37] Shimizu Y., Inoue A., Tomari Y., Suzuki T., Yokogawa T., Nishikawa K. and Ueda T.(2001). Cell-free translation reconstituted with purified components. Nat Biotechnol 19(8): 751-755. 11479568 10.1038/90802

[38] Simsek D. and Barna M.(2017). An emerging role for the ribosome as a nexus for post-translational modifications. Curr Opin Cell Biol 45: 92-101. 28445788 10.1016/j.ceb.2017.02.010 PMC5731828

[39] Svitkin Y. V. and Agol V. I.(1978). Complete translation of encephalomyocarditis virus RNA and faithful cleavage of virus-specific proteins in a cell-free system from Krebs-2 cells. FEBS Lett 87(1): 7-11. 204520 10.1016/0014-5793(78)80121-5

[40] Svitkin Y. V. and Sonenberg N.(2004). An efficient system for cap- and poly(A)-dependent translation in vitro. Methods Mol Biol 257: 155-170. 14770004 10.1385/1-59259-750-5:155

[41] Svitkin Y. V. and Sonenberg N.(2007). A highly efficient and robust in vitro translation system for expression of picornavirus and hepatitis C virus RNA genomes. Methods Enzymol 429: 53-82. 17913619 10.1016/S0076-6879(07)29004-4

[42] Taoka M., Nobe Y., Yamaki Y., Sato K., Ishikawa H., Izumikawa K., Yamauchi Y., Hirota K., Nakayama H., Takahashi N., .(2018). Landscape of the complete RNA chemical modifications in the human 80S ribosome. Nucleic Acids Res 46(18): 9289-9298. 30202881 10.1093/nar/gky811 PMC6182160

[43] Thoma C., Ostareck-Lederer A. and Hentze M. W.(2004). A poly(A) tail-responsive in vitro system for cap- or IRES-driven translation from HeLa cells. Methods Mol Biol 257: 171-180. 14770005 10.1385/1-59259-750-5:171

[44] Trainor B. M., Pestov D. G. and Shcherbik N.(2021). Development, validation, and application of the ribosome separation and reconstitution system for protein translation in vitro. RNA 27(12): 1602-1616. 34452990 10.1261/rna.078852.121 PMC8594471

[45] Wang C., Han B., Zhou R. and Zhuang X.(2016). Real-time imaging of translation on single mRNA transcripts in live cells. Cell 165(4): 990-1001. 27153499 10.1016/j.cell.2016.04.040 PMC4905760

[46] Witherell G.(2001). In vitro translation using HeLa extract. Curr Protoc Cell Biol Chapter 11: Unit 11.8. doi: 10.1002/0471143030.cb1108s06. 18228313

[47] Wu B., Eliscovich C., Yoon Y. J. and Singer R. H.(2016). Translation dynamics of single mRNAs in live cells and neurons. Science 352(6292): 1430-1435. 27313041 10.1126/science.aaf1084 PMC4939616

[48] Yan X., Hoek T. A., Vale R. D. and Tanenbaum M. E.(2016). Dynamics of translation of single mRNA molecules in vivo. Cell 165(4): 976-989. 27153498 10.1016/j.cell.2016.04.034 PMC4889334

[49] Yokoyama T., Machida K., Iwasaki W., Shigeta T., Nishimoto M., Takahashi M., Sakamoto A., Yonemochi M., Harada Y., Shigematsu H., .(2019). HCV IRES captures an actively translating 80S ribosome. Mol Cell 74(6): 1205 12141214.e8. 31080011 10.1016/j.molcel.2019.04.022

